# A Tension Sensor Array for Cable-Driven Surgical Robots

**DOI:** 10.3390/s24103156

**Published:** 2024-05-16

**Authors:** Zhangxi Zhou, Jianlin Yang, Mark Runciman, James Avery, Zhijun Sun, George Mylonas

**Affiliations:** 1 The Hamlyn Centre, Institute of Global Health Innovation, Imperial College London, London W2 1PF, UK; zhangxi.zhou22@imperial.ac.uk (Z.Z.); jianlin.yang@imperial.ac.uk (J.Y.); m.runciman@imperial.ac.uk (M.R.); james.avery@imperial.ac.uk (J.A.); 2 State Key Laboratory of Mechanics and Control of Aerospace Structures, Nanjing University of Aeronautics and Astronautics, Nanjing 210016, China; meezjsun@nuaa.edu.cn

**Keywords:** cable-driven parallel robot (CDPR), cable tension sensing, sensor design, palpation, force estimation

## Abstract

Tendon–sheath structures are commonly utilized to drive surgical robots due to their compact size, flexibility, and straightforward controllability. However, long-distance cable tension estimation poses a significant challenge due to its frictional characteristics affected by complicated factors. This paper proposes a miniature tension sensor array for an endoscopic cable-driven parallel robot, aiming to integrate sensors into the distal end of long and flexible surgical instruments to sense cable tension and alleviate friction between the tendon and sheath. The sensor array, mounted at the distal end of the robot, boasts the advantages of a small size (16 mm outer diameter) and reduced frictional impact. A force compensation strategy was presented and verified on a platform with a single cable and subsequently implemented on the robot. The robot demonstrated good performance in a series of palpation tests, exhibiting a 0.173 N average error in force estimation and a 0.213 N root-mean-square error. In blind tests, all ten participants were able to differentiate between silicone pads with varying hardness through force feedback provided by a haptic device.

## 1. Introduction

Robot-assisted minimally invasive surgery has gained increasing popularity due to its numerous advantages, such as reducing pain, lowering the risk of incision infection, and accelerating recovery [1]. Tendon-sheath mechanisms (TSMs) are the most prevalent mechanisms for actuating surgical tools, owing to their small size, high strength, and flexibility.

Cable-driven robots are common in surgical applications. Mylonas et al. proposed the Cyclops concept, a cable-driven parallel robot (CDPR) offering bimanual instrument triangulation and high force transmission over a large workspace, which was further developed to perform Endoscopic Submucosal Dissection (ESD) [2]. Liu et al. [3] developed a foldable robot hand for robot-assisted laparoscopic surgery, with three snake-like continuum fingers equipped with force sensors at the fingertips to allow sensing tissue stiffness using palpation. Additionally, Pedram et al. [4] proposed a novel needle path planning algorithm based on a cable-driven surgical robot platform, which enables autonomous suturing by bimanual operation.

Due to operational and safety requirements, cable tension perception plays an important role in cable-driven robots for surgery. Cable tension sensing can be used to monitor and control the force exerted on the patient to prevent excessive force that may cause tissue damage [5]. If the detected cable tension is abnormal, such as in the case that some cable is stuck, the robotic device could automatically stop to prevent surgical instruments from losing control and protect patients from being injured. Additionally, precise cable tension sensing can provide haptic feedback, enabling surgeons to differentiate between normal, cancerous, or scarred tissue, or detect hidden tumors based on sensing localized differences in tissue stiffness [6,7,8].

Recently, several sensors have been designed for tissue palpation and haptic feedback during minimally invasive surgery, including Fiber Bragg Grating (FBG) sensors [9,10], capacitive sensors [11], and tactile sensors with feedback mechanisms [12,13]. Additionally, there are also cable-driven robots equipped with force sensors [6,14,15,16,17] to detect cable tension close to the actuators (i.e., driving motors), but their force estimation would be affected by friction.

Tension sensing for cable-driven robots can be broadly divided into two categories, short-distance and long-distance, according to cable length. Short-distance sensing is less affected by friction due to a short force transmission distance and a small cable bending angle, which is commonly integrated with surgical robots such as the DaVinci [3,15,16,17]. 

In contrast, long-distance cable tension sensing is greatly affected by friction due to large bending angles and long force transmission distances, posing greater challenges in force estimation. In previous studies, force sensing at the distal end of cable-driven robots was commonly implemented by two approaches. One approach is integrating sensors into the end-effector to directly measure the external force [3,14]. However, it is quite challenging to integrate tactile sensors into the distal end of a surgical tool due to the confined workspace. Another approach is estimating the external force by mounting sensors at the proximal side (close to the motors actuating the cables). For example, sensors like strain gauges, load cells, or motor parameters (e.g., motor current) were used to estimate the force distribution or the state of the end-effector [18,19]. In these cases, cable tension is normally estimated based on mathematical models. In [20], a Dahl friction model was employed to model pulley-bearing friction and predict tension during rapid transitions in a CDPR. The predicted tension was then utilized to simulate tension profiles at varying velocities. Do et al. [21] introduced a new friction model approach, leveraging cable-sheath velocity and acceleration to accurately estimate friction in sliding and pre-sliding states. Moreover, Kraus et al. [22] applied Coulomb and Dahl friction models to measure and compensate for pulley friction, thus determining the cable tension. 

However, accurate friction estimation is as challenging as it is important, especially in cable-driven flexible endoscope robots, which need to navigate through confined spaces and narrow and long human cavities, such as the colon. Force estimation by the model-based methods described above is difficult to implement, as the shape of the long cable-sheath mechanisms is difficult to measure to obtain the model parameters.

Monitoring cable tension is critical for safe operation, while external force information provides valuable force feedback to surgeons. In this paper, a novel tension sensor array is proposed for cable-driven surgical robots. The tension sensor array is equipped with a miniature-sized Force Sensing Resistor (FSR), which allows it to be mounted at the robot’s distal end. The array’s force estimation is less affected by friction and it offers more accurate measurements of cable tension and the ability to estimate the force acting on the robot tip. We develop a force estimation method based on the Capstan equation to evaluate the tension sensing performance on a single-cable platform and use it to conduct a series of palpation tests with the robot. During the test, the robot demonstrates good external wrench sensing through various force compensation strategies. After mapping the estimated external wrench to a haptic device, participants were able to distinguish objects of different stiffness using master–slave control based solely on force feedback.

## 2. Robot and Sensor Design

### 2.1. Robot System Overview

**Figure 1:**
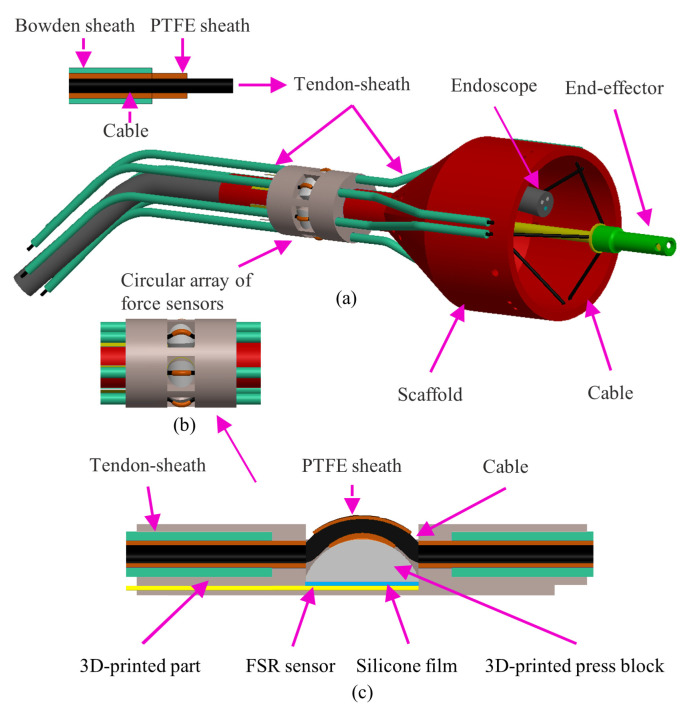
Overall structure of the robot. (**a**) Robot overview; (**b**) FSR circular sensor array; (**c**) Single FSR sensor.

Figure 1 shows the robot concept design. In Figure 1a, the robot’s primary feature is a scaffold, desired to be soft, deployable, and variable in stiffness, similar to the structure presented in [23]. The scaffold is manufactured using a laser welding system which selectively seals together thermoplastic sheet laminates, enabling the creation of airtight chambers. This allows it to be folded into a small volume while deflated, but once the chambers are inflated and pressurized, the scaffold’s structure can be expanded into a prismatic shape and its stiffness significantly increases. Moreover, by regulating the inflation pressure, we can controllably change its overall stiffness.

**Figure 2:**
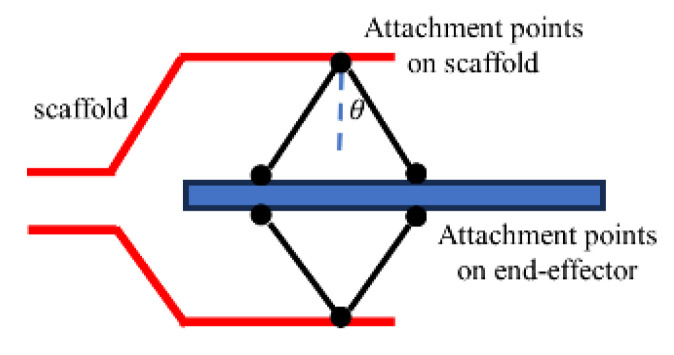
Diagram of CDPR’s cable bending angle θ.

The scaffold is attached to an endoscope. Since this study is focused on cable tension sensing, we use a 3D-printed rigid scaffold to validate the sensor design. The driving mechanism, a tendon–sheath system, is composed of a Bowden tube, a PTFE tube to reduce friction, and a cable. In Figure 1b, a circular sensor array, equipped with six FSR sensors, shown in Figure 1c, is mounted near the scaffold to measure cable tension. Figure 2 shows an example of a CDPR configuration where the bending angle θ will change as the end-effector moves.

In the envisioned surgical scenario, the robot’s operational process involves a soft inflatable scaffold delivered as an over-the-scope attachment through the intestines, which is then inflated and expanded internally to prepare for surgery. Sensor placement is crucial; proximal placement results in significant friction-related perception interference (e.g., due to cable bending), while distal placement behind the scaffold lessens this effect and facilitates integration with standard endoscopes due to the closer proximity of the sensors to the end-effector.

**Figure 3:**
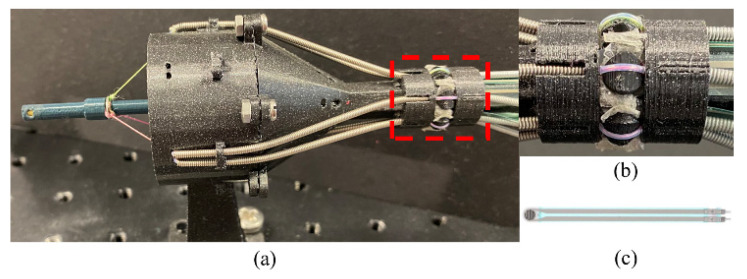
Robot prototype. (**a**) Overview of the robot; (**b**) the circular array of FSR sensors; (**c**) Force-Sensing Resistors.

**Figure 4:**
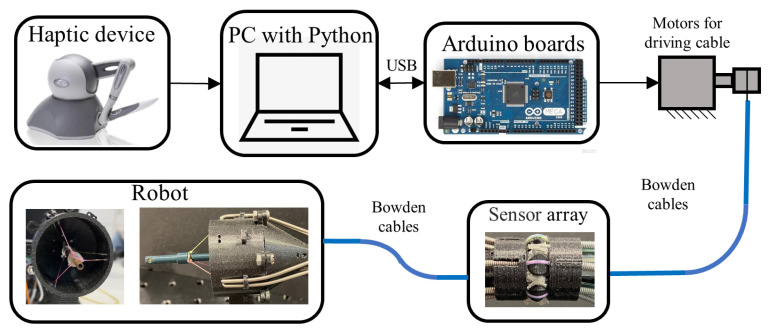
The robot system.

Figure 3 shows the robot prototype. The circular array in Figure 3b has a 16 mm outer diameter and 20 mm length, while Figure 3c shows the chosen FSR (RP C5LT LF5) with 5–600 g capacity, 5 mm outer diameter of the sensing head, and 0.2 mm thickness. Figure 4 depicts the complete robot system. A laptop is linked to the Arduino Mega2560 board, which controls NEMA17 stepper motors for driving the cable. The Bowden cables originate from the stepper motors, pass through the tension sensor array, and establish a connection with the robot prototype. A Geomagic Touch haptic device (3D Systems, Littleton, CO, USA) is used to control the robot. The tension array, with an outer diameter of 16 mm, is deemed adequately compact to navigate through the colon, which typically has a diameter between 26 and 45 mm [24].

### 2.2. Sensor Design and Calibration

Considering the size constraints of endoscopes, the sensor array must be compact. Among the various force sensors used in surgical robots—such as strain gauges, piezoelectric sensors, capacitive sensors, and FBG [25,26]—the piezoresistive FSR sensor is selected for its thinness, small size, ease of integration with compact structures, and affordability.

Each FSR sensor’s arrangement in Figure 1 involves a cable pressing against a 3D-printed block, which then applies pressure to the FSR sensor. A thin silicone film is placed between the FSR sensor and the press block to ensure full contact and optimal sensor sensitivity.

**Figure 5:**
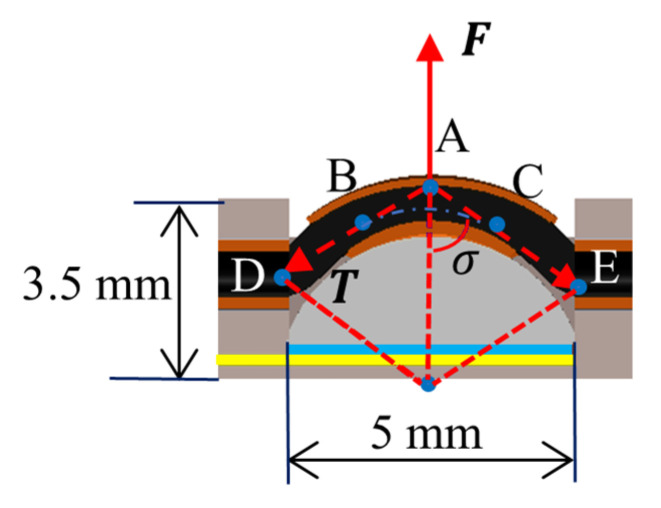
Force diagram of a single cable sensor.

Force analysis was performed on the sensor array to determine the correlation between the tension and the pressure recorded by the force sensor. Figure 5 illustrates the force diagram of a single sensor unit. A cable threads through the unit, and when tightened, it applies a downward force on the force sensor, altering the readings from the FSR.

In this context, F denotes the reaction force resulting from the cable’s pressure. The tension T is represented by the line segments AE and AD, with point A being the force application point, and points B and C serving as the tangent points between the tension and the arc. The angle σ is the angle formed between the tension and the vertical line. The relationship between F and T is influenced by the dimensions (such as σ and height) of the press block and the 3D-printed part. Equation (1) shows an ideal relationship regarding the geometric dimensions, while the actual relationship between F and T will be measured and derived based on experimental data as follows.

(1) $$\frac{F}{2T}=\cos\sigma$$

**Figure 6:**
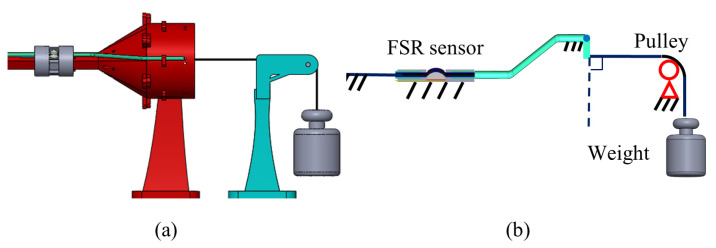
Sensor calibration platform. (**a**) Schematic diagram. (**b**) Skeleton diagram.

Figure 6 shows the sensor calibration platform. The calibration process involves measuring sensor readings under both loading and unloading conditions. Hysteresis is observed due to friction in the tendon–sheath mechanism. The cable, threading through the robot scaffold, is connected to a weight via a pulley. The cable tension is assumed to be equal to the weight. The calibration process includes both loading and unloading due to the differences in the direction of friction, which yields varying sensor readings. The loading and unloading process was repeated three times and the average values were recorded.

**Figure 7:**
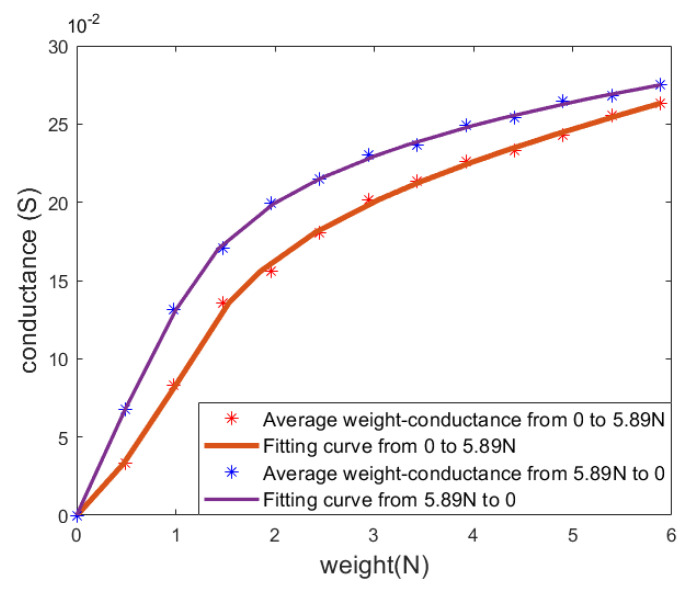
An example of the relationship between measured sensor readings and tension in a single cable. The red and purple curves are obtained through loading and unloading process, respectively.

Figure 7 depicts the correlation between the measured sensor readings (conductance) and the tension of a single sensor during the loading and unloading of the weights, while the fitted curves are obtained by quintic polynomial fitting:

(2) $$P\left(x\right)=a\prod_{i=1}^{5}\left(x-x_{i}\right)$$

where x is the weight, $P\left(x\right)$ is the conductance, and a and $x_{i(i=1,\ldots ,5)}$ are the leading coefficient and the five roots of the polynomial, respectively.

The red and blue points in Figure 7 are the sensor readings and tension (i.e., gravity acting on the weight) measured by gradually adding and removing weight from 0 to 600 g and from 600 to 0 g, in steps of 50 g. The reason that loading and unloading readings at the 600 g weight (5.89 N) are different is that the unloading 5.89 N reading is acquired when the weight decreases from 650 g (6.38 N) to 600 g (5.89 N), whereas the loading 5.89 N reading is obtained when the weight increases from 550 g (5.40 N) to 600 g (5.89 N). 

## 3. Force Compensation of a Single Cable

In this section, we present two tests. The first test serves two purposes: one is used to demonstrate the difference in estimated tension between measurements at the proximal and distal ends, when bending angle θ changes; the second is to show the effect of the Capstan equation in improving the accuracy of the modelled tension. The second test is used to validate the modelled tension under variable speed motion.

**Figure 8:**
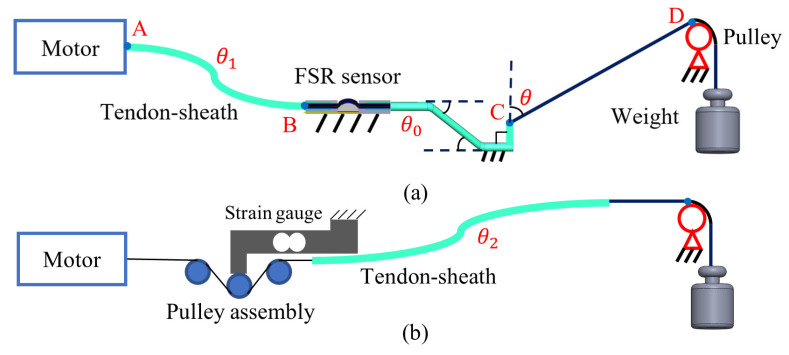
Single cable test platforms. (**a**) FSR sensor at the distal end; (**b**) strain gauge at the proximal end.

The first test is performed using the platforms shown in Figure 8. Figure 8a illustrates a platform designed to evaluate the friction compensation effect of a sensor operating on a single cable. If the force compensation proves effective on a single cable, the same strategy can be implemented on each cable of the robot. Point A in Figure 8a is referred to as the proximal end, while point C is the distal end. The cable can be considered as three segments: AB, BC, and CD:

- The AB segment represents the cable connecting the robot and the motor. Its bending angle, $\theta_{1}$, varies as it progresses through the intestine.
- The BC segment forms part of the robot scaffold structure, with its bending angle $\theta_{0}$ remaining constant.
- The CD segment signifies the cable that pulls the end-effector from the scaffold. Its bending angle, denoted as θ, is calculated through robot kinematics during operation.

The platform maintains the cable bending angles $\theta_{0}=140{}^{\circ}$ and $\theta_{1}=360{}^{\circ}$, mirroring the robot configuration in Figure 1. The angle $\theta_{0}$ refers to the cumulative angle from point B to point C, which includes the sum three angles marked with dotted lines and arcs in Figure 8a.

The bending angle θ can be adjusted to 0°, 30°, 60°, and 90°. Point D is designated for weight addition.

To illustrate the tension estimation difference between measurements at the proximal and distal ends, this test takes place in two scenarios: one where the FSR sensor is placed at the robot’s distal end with the platform in Figure 8a, and the other where a strain gauge is positioned at the proximal (motor) end with the platform in Figure 8b, both with identical cable bending angles where $\theta_{2}=\theta_{1}+\theta_{0}+\theta$. Before the test, the sensor is pre-tensioned with weights, and then the motor is set to move back and forth, while the cable tension is recorded either at the distal end by the FSR or at the proximal end by the strain gauge. During the motor motion, the weight remains constant; thus, the recorded force change is primarily due to the friction between the moving cables and their sheath. Therefore, the experiment highlights the extent to which the two setups depicted in Figure 8 are affected by friction.

**Figure 9:**
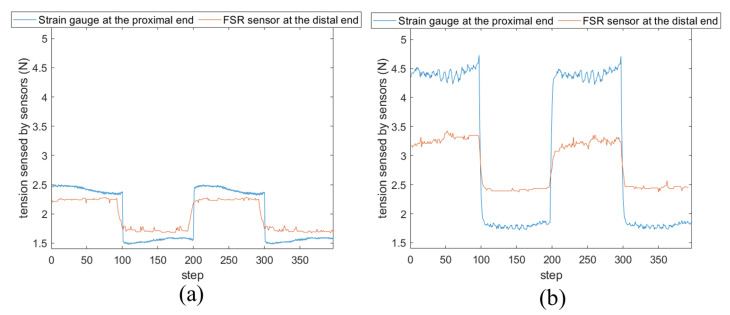
Comparison of measured force changing when putting the sensors at the proximal side and the distal side. (**a**) A comparison when θ is 0° and the pre-tension is 1.96 N; (**b**) a comparison when θ is 30° and the pre-tension is 2.94 N.

Figure 9 shows the comparison of perceived force changes. In Figure 9a, with a cable bending angle θ of 0° and a pre-tension of 1.96 N (i.e., 200 g weight from the pulley), the FSR sensor at the distal end registers a change of 0.54 N (from 2.25 N to 1.71 N), while the strain gauge at the proximal end records a change of 0.98 N (from 2.48 N to 1.50 N). In Figure 9b, where the cable bending angle θ is 30° and the pre-tension is 2.94 N (300 g weight at pulley), the FSR sensor at the distal end shows a change of 0.93 N (from 3.32 N to 2.39 N), while the strain gauge at the proximal end indicates a change of 2.69 N (from 4.43 N to 1.74 N).

The results demonstrate that, firstly, the sensor’s reading variation range increases with the bending angle and tension. Secondly, the perceived force variance of Figure 8a is smaller than that of the platform of Figure 8b with the same cable bending angle, which demonstrates that the FSR sensor positioning at the robot’s distal end can be less influenced by friction, which is a key advantage of this design. 

To improve the accuracy of the modelled tension, we performed more tests using the platform shown in Figure 8a to compensate for the friction. To this end, the Capstan equation is implemented to estimate the modelled tension between point C and the effector.

**Figure 10:**
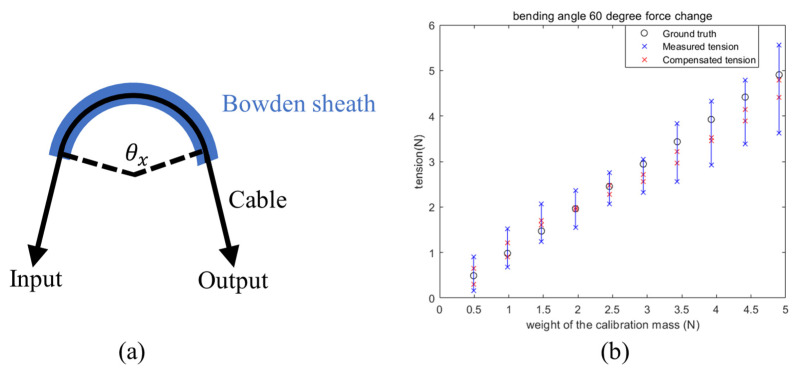
Force compensation effect of the single cable testing using the setup shown in Figure 8a. (**a**) Robotic Capstan equation model. (**b**) The comparison of force stability before and after applying the Capstan equation.

The Capstan equation outlines how force changes when moving a cable around a Capstan, which explains the interaction relationship between tension and friction. Figure 10a shows the Capstan equation model utilized for the robot. The bending formed by the Bowden sheath, owing to its inherent rigidity, exhibits a similar function to the Capstan.

(3) $$T_{load}=T_{hold}e^{\mu \left(\theta_{x}\right)}$$

where $T_{load}$ and $T_{hold}$ are the input and output forces, respectively. When the platform is loaded as shown in Figure 8a, the input force is the modelled tension, while the output force is the weight’s gravity. The input and output forces are reversed during unloading. μ is the friction coefficient, and $\theta_{x}$ is the sum of cable bending angle θ and $\theta_{0}$.

The results in Figure 10b were collected by varying the tension (weight of the calibration mass) from 0.49 N to 4.9 N (equivalent to a weight range of 50–500 g) for a 60° bending angle θ. For each tension, the motor moved forward and backward three times. In other words, by varying the weights and conducting repeated experiments, graphs similar to Figure 9 were obtained, while Figure 10b resulted from statistically analyzing all their variation ranges. Figure 10b demonstrates the results that compare the sensor’s reading changes without compensation and after compensation using the Capstan equation.

The results indicate that for a constant weight, the change in the sensor’s modelled force is smaller after applying the Capstan equation for compensation. This suggests that, with the Capstan equation, tension sensing becomes more stable when the motor’s direction of movement changes.

**Figure 11:**
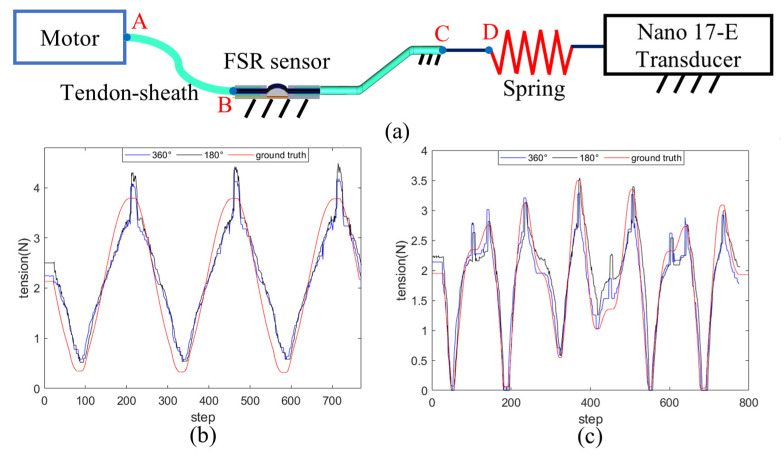
Verification of a single cable force compensation. (**a**) An overview of the platform. (**b**) Force estimation results with motor moves following a sinusoidal function. (**c**) Force estimation results with motor moves following a superposition of two sinusoidal functions.

The second test verifies the force compensation effect of a single cable, as shown in Figure 11. Figure 11a shows the test platform, with segments BC and CD having the same size and bending angle as the tendon–sheath assembly of the actual robot. The cable starts from the motor side, passes through the FSR sensor, and is linked to a spring connected to a Nano 17-E Force/Torque Transducer (F/T sensor). The cable pulls the spring over different distances to generate different cable tensions. Subsequently, the motor moves at variable speed to pull the cable and spring. Throughout this process, the F/T sensor captures the ground truth of tension variations. The modelled tension is calculated using the Capstan equation. Figure 11b,c show a comparison between the measured tension and the ground truth when the AB segment in Figure 11a bends 360° and 180°. Figure 11b,c indicate the following:

- The force measured by the FSR aligns well with the ground truth, suggesting that the bending angle compensation increases the accuracy of the modelled force.
- When the motor’s direction or speed changes rapidly, the FSR readings will experience a sudden shift before quickly returning to normal. This phenomenon is attributed to hysteresis and changes in the direction of friction.
- The results for 360° and 180° bending angles of section AB are almost identical. This means that when applying the Capstan equation for compensation, the cable bending angle is only related to the BC and CD sections, and there is no need to consider the AB section.

The independence of tension sensing from the AB segment’s cable bending angle is an advantage of the design, as when the robot moves inside a human lumen, the bending angle $\theta_{1}$ at AB will change greatly. According to the Capstan equation, this angle would largely affect the magnitude of force compensation. Since there is no need to take the bending angle at AB into account, only the angles at the BC and CD segments (θ and $\theta_{0}$) need to be considered, which will considerably simplify the operation.

## 4. Force Compensation Strategy of the Robot

Friction within the tendon–sheath during motion can significantly affect the sensor’s force perception, leading to substantial errors. To measure the actual force exerted by each cable during the operation of the robot more accurately, we adopted the following force compensation strategy:

Step 1. Using the Capstan equation to compensate for all six cables.

**Figure 12:**
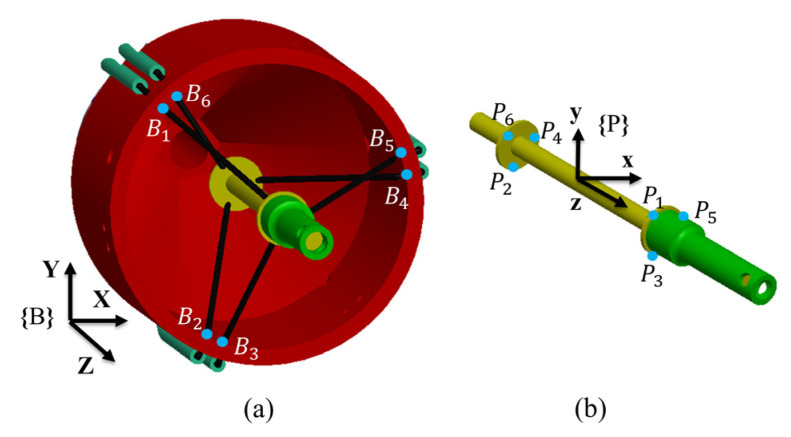
Frames of {B} and {P}. (**a**) Frame {B} on the scaffold. (**b**) Frame {P} on the end-effector.

The calculation of each cable’s bending angle is essential and is determined based on the robot’s kinematics. Subsequently, the Capstan equation is employed to compensate for the measurement force of each cable. Figure 12 illustrates the moving frame {P} located on the end-effector and frame {B} positioned on the scaffold, which is similar to [2]. The initial state of the end-effector in Figure 12 indicates that all six cables have the same length.

The vector $l_{i}$ along the cable is determined by the vector from the point on scaffold $B_{i}$ to the corresponding attachment point on the end-effector $P_{i}(i=1,2,\ldots ,6)$.

(4) $$l_{i}=B_{i}P_{i}$$

The position of each $p_{i}$ on the end-effector can be represented in the base frame as follows:

(5) $$p_{i}=p+Rr_{i}$$

The vector $r_{i}$ denotes the position of the attachment point expressed in frame {P}, p concerns the expression of end-effector’s center of mass in the base frame, and R is the rotation matrix mapping moving frame {P} to base frame {B} using the Z-Y-X Euler angles convention. 

The cable bending angle which corresponds to the CD segment in Figure 8a can be calculated as

(6) $$\theta =90{}^{\circ}-arccos\frac{{v_{i}\cdot l}_{i}}{\left|v_{i}\right|\cdot \left|l_{i}\right|}$$

where $v_{i}$ is the normal vector of the plane formed by the circle constituted by the six points $B_{i}(i=1,2,\ldots ,6)$. 

Then, the measured force can be compensated using the Capstan equation:

(7) $$T_{load}=T_{hold}e^{\mu \left(\theta +\theta_{0}\right)}$$

where $\theta_{0}$ is the cable bending angle of the robot scaffold structure (corresponding to the BC segment in Figure 8a). The input force and output force are identified by the increase and decrease in the cable length (direction of cable movement). For example, during the robot operation, if the length of one cable increases, it is the unloading process, and the input force is the FSR modelled force, and vice versa.

Step 2. Calculating the external wrench based on the robot structure matrix.

According to [27], the force equilibrium is given as

(8) At+f=0

where $f=[F_{p},\tau_{p}]$ is the external wrench and torques acting on the end-effector, ***t*** is the tension of each cable ${t=[\begin{array}{cc} \begin{array}{ccc} t_{1} & t_{2} & t_{3} \end{array} & \begin{array}{ccc} t_{4} & t_{5} & t_{6} \end{array}] \end{array}}^{T}$, and ***A*** is the structure matrix of the system, given by

$$A=\left[\begin{array}{ccc} -\frac{l_{1}}{l_{1}} & \cdots & -\frac{l_{6}}{l_{6}}\\ -p_{1}\times \frac{l_{1}}{l_{1}} & \cdots & -p_{6}\times \frac{l_{6}}{l_{6}} \end{array}\right]$$

where $l_{i}(i=1,2,\ldots ,6)$ is the modulus length of vector $l_{i}$.

Step 3. External wrench friction compensation during end-effector direction changes.

**Figure 13:**
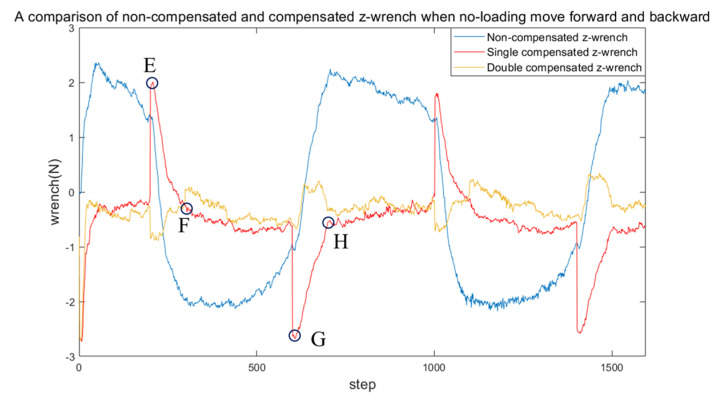
A comparison of non-compensated and compensated z-wrench. Segments EF and GH represent the sudden change in wrench when the end-effector turns.

In Figure 13, the EF and GH segments represent the sections that undergo a degree of compensation during the forward and backward movements, respectively. Polynomial fitting is utilized to fit the EF and GH segments, to compensate for abrupt changes in the sensed external force during end-effector direction changes. The variation in the sensed external force (without payload) is illustrated as the effector oscillates in 20 mm distance twice along the *z*-axis at an approximate speed of 0.5 mm/s. The graph presents three scenarios: without any compensation, with single compensation, and with double compensation applied by the compensation strategy.

As depicted by the blue line in Figure 13, without any compensation, the sensor readings exhibit instability during no-load motion. After applying the first round of compensation, a sudden change in the reading is observed upon turning, although the readings remain stable in other areas. When a second round of compensation is applied, the abrupt change observed during turning is diminished, resulting in stable readings throughout the movement.

## 5. Robot Wrench Force Compensation Effect and Palpation Tests

**Figure 14:**
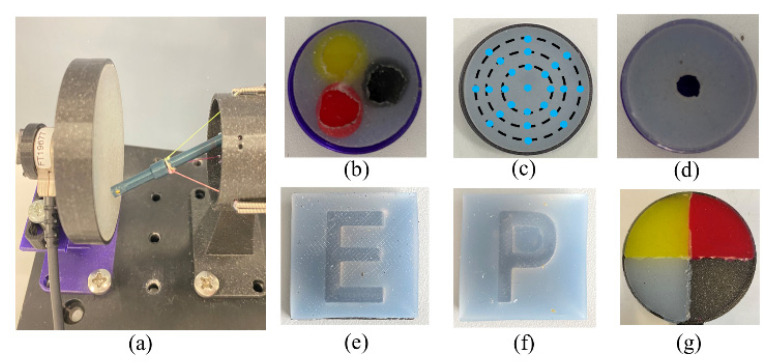
Silicone pads used for palpation with 6 mm thickness. (**a**) The setup for the palpation test. (**b**–**g**) The silicone pads used for different palpation tests. Size: (**b**) 27 mm diameter; (**c**,**g**) 60 mm diameter; (**d**) 27 mm diameter with an 8 mm diameter hole in the middle; (**e**,**f**) 30 × 30 mm.

The following section builds on the developed methodologies to complete a series of palpation tests with the robot. Figure 14 shows the various silicone pads used for the different palpation tests. During the palpation process, the end-effector is moved to contact the target area to poke forward. Throughout this procedure, tension exerted on each cable is recorded by the FSR sensor array. Real-time calculations are performed to determine the external wrench applied to the end-effector. The Nano17-E Transducer in Figure 14a is mounted at the back of the silicone pad for obtaining wrench ground truth data. In Figure 14b–f, the gray/white silicone pads are made of Ecoflex 00–20 with 00–20 Shore hardness. In Figure 14b, the red, yellow, and black pads are made of Dragon Skin–30, Ecoflex 00–30, and Ecoflex 00–10, with a Shore hardness of A–30, 00–30, 00–10, respectively. In Figure 14g, the yellow, red, white, black pads are made of Ecoflex–50, Dragon Skin–30, Ecoflex–20, and PLA filaments (rigid, 3D-printed), respectively.

### 5.1. Results of Palpation

The following three tests delineate the performance of the tension array. The first test, stiffness detection, shows the robot’s potential in discerning tumors with differing stiffness from normal tissue. The second test demonstrates the robot’s general palpation performance along the x, y, and z axes. The final scanning test illustrates the robot’s performance in identifying over an area with varying stiffness.

Test 1. Palpation—stiffness detection

**Figure 15:**
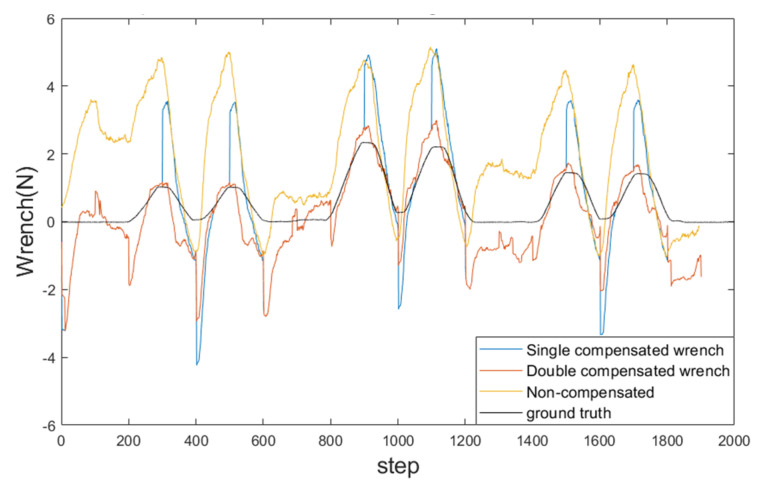
Results of touching three regions with different stiffnesses, which is the comparison between no compensation, single compensation, double compensation, and ground truth.

Figure 14b features a silicone block used to simulate three regions with different stiffness levels in the following order: red >> yellow > black. As the robot moves for palpation at an estimated speed of 0.5 mm/s, the end-effector makes contact with these three regions vertically. Figure 15 shows the external wrench sensed during palpation under different force compensations. The numerical results are presented in Table 1. For each poke, the measured result is obtained from the deepest point along the *z*-axis. As evident in Figure 15, after applying two rounds of compensation, the sensed external wrench becomes smoother, indicating the effectiveness of the compensation strategy in enhancing the stability of the force measurements. 

As indicated in Table 1, the perceived external force, after applying two rounds of compensation, is very close to the actual value obtained with the F/T sensor. In contrast, the results without compensation deviate significantly from the ground truth and fail to distinguish the stiffness differences. This experiment effectively highlights the performance disparity between the system with and without force compensation.

Test 2. Palpation—x-, y-, z-direction test

**Figure 16:**
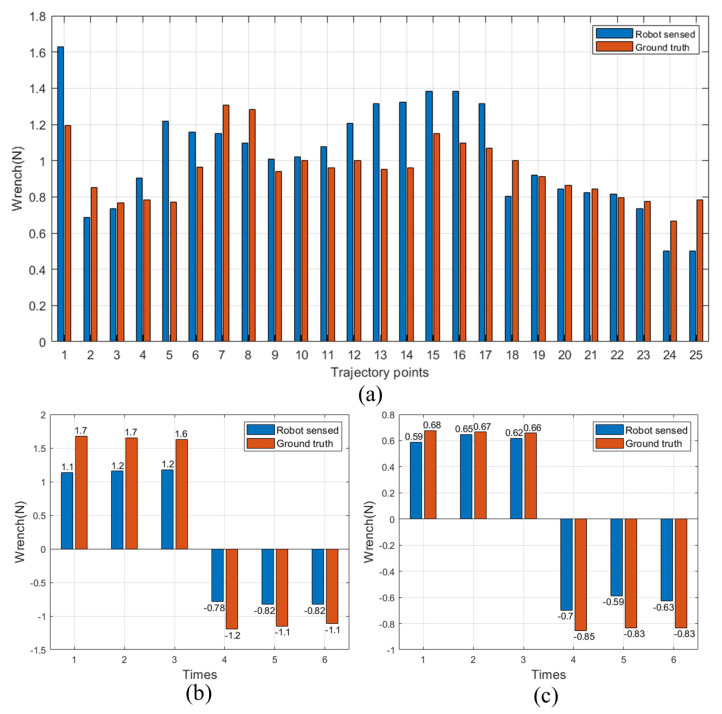
Palpation test results: (**a**) z-direction (**b**) x-direction. (**c**) y-direction.

The robot was used to perform palpation tests in the x-, y-, and z-directions. Figure 14c illustrates the silicone pad and poking points employed in the z-direction palpation tests, comprising 25 trajectory points corresponding to the 25 columns of results in Figure 16a. Trajectory points 1, 2–9, 10–17, and 18–25 indicate that the robot pokes the origin, the 8 mm diameter circle, the 16 mm diameter circle, and the 24 mm circle for palpation test, respectively. The whole test was repeated 5 times. The average error of the robot operation is determined to be 0.173 N, with a root-mean-square error of 0.213 N.

In Figure 14d, the silicone pad designed for evaluating wrench performance along the x and y axes is depicted. The robotic system engages with the silicone pad by inserting the effector’s end into the hole in the middle to make contact with the pad three times in both positive and negative directions along the x and y axes. Figure 16b,c present the outcomes obtained when the tip of the effector moves along the x and y axes. The measurement results are extracted from the deepest points along the x and y axes. The average error is calculated to be 0.268 N, with a root-mean-square error of 0.321 N.

For comparison, the results of our tension sensor array with other related cable-driven surgical robots are presented in Table 2.

Test 3. Palpation—letter “E” and “P” letter scanning and identifying

**Figure 17:**
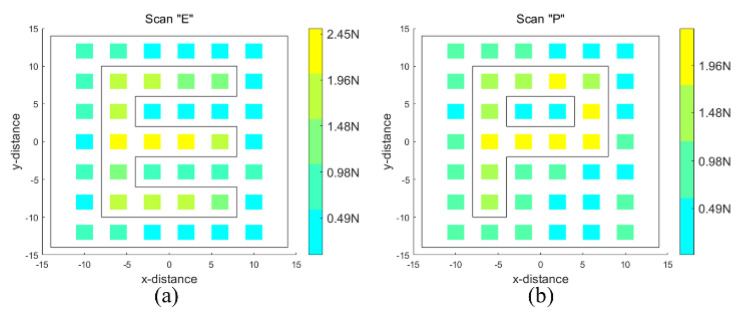
Reconstructed image of the letter “E” and “P”. (**a**) Scan “E” (**b**) Scan “P”.

Figure 14e,f show the letters used for scanning. The rigid letter pattern is covered with silicone. The robot scanned an array of 6 × 7 points with the end-effector poked 5 mm deep. Figure 17a,b combine the external wrench sensed by the robot at each point to reconstruct the letters “E” and “P”. It shows the relative position of the poked points and the scanned letters “E” and “P”, as well as the reconstructed image represented by color gradients. The scanning results indicate that the robot is able to distinguish between rigid letter patterns and soft silicone, demonstrating the ability of the robot to also sense through palpation tumors hidden under the tissue surface.

### 5.2. Blind Tests

In the blind test, ten participants were invited to interact with the four areas shown in Figure 14g using the robot controlled via a haptic device. The aim was to evaluate the stiffness of these areas based on the force feedback from the device. The four areas of stiffness are ranked as follows: “Rigid (black)” > “Dragon Skin 30 (red)” > “Ecoflex 50 (yellow)” > “Ecoflex 20 (white)”. The areas were randomly rearranged for each test, and all forces generated in the negative direction of the *z*-axis were set to zero to prevent misjudgment and interference.

**Figure 18:**
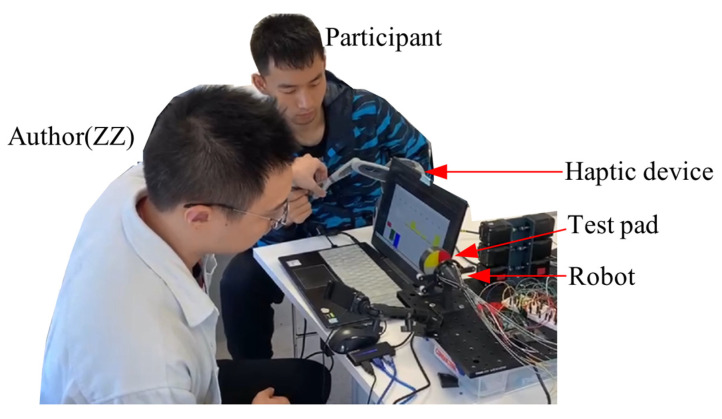
Experimental setup and conditions during the blind test.

The experimental setting is shown in Figure 18. Ten participants, eight male and two female, aged around 25 years old with an engineering background, took part in this blind test. The study coordinator (author ZZ) used the haptic manipulator handle to bring the effector just over one of the silicone pads. Participants then pushed the handle forward and were asked to memorize the perceived hardness of the probed area. The coordinator moved the effector to the next area and instructed the participants to continue until all four areas were tested. The location and order of the regions were randomized. During the procedure, participants could not see where the effector was or whether it was in contact with the silicone film, as the back side of the silicone test pad prevented participants from seeing the robot’s effector (see Figure 18). For areas with small differences in hardness, participants were allowed to conduct multiple pokes. 

During the testing process, the author initially choose two areas randomly for participants to palpate, referred to as Zone 1 and Zone 2. Participants compared the hardness of Zones 1 and 2. Next, another area, Zone 3, was randomly selected for palpation, and participants compared its hardness relative to Zones 1 and 2, determining the order of hardness among Zones 1, 2, and 3. Subsequently, participants moved to Zone 4, comparing its hardness relative to Zones 1–3, thus establishing the overall ranking.

For instance, if Zones 1, 2, 3, and 4 are randomly represented by the colors red, black, yellow, and white, respectively, the initial comparison might result in the hardness ranking: Zone 1 < Zone 2. During the second comparison, if Zone 2 > Zone 3 and Zone 1 > Zone 3, the final ranking would be Zone 2 > Zone 1 > Zone 3. The same process is repeated for Zone 4, comparing its hardness to Zones 1, 2, and 3, respectively, and then determining the final hardness ranking. Throughout the test, if a participant was uncertain about the hardness of two areas, the author moved the robot to the respective areas for multiple palpations upon the participant’s request.

When converting these rankings into numerical representations, participants were instructed to rate the hardness of four areas by assigning the hardest zone a number between 8 and 10 and the softest zone a number between 1 and 3. Then, based on their remembered hardness, they would write down the relative numbers for comparison. 

Table 3 presents the results of the blind test, with numbers indicating the perceived hardness. The results reveal that the hardness of the four areas is ranked as black > red > yellow > white, which aligns with reality. 

In addition to the blind test, the participants also tried to use the haptic device with vision feedback to test and distinguish four areas of different stiffness. The results showed that everyone was able to correctly distinguish and rank the stiffness of different areas under vision feedback and haptic force feedback.

Through the above blind tests, the performance of the tension array for palpation is demonstrated. A critical aspect is that, unlike other tactile sensors which require an additional/separate end-effector, the sensor presented here can be incorporated in existing surgical tools, such as a grasper, making palpation feasible during endoscopic surgery.

## 6. Discussion and Conclusions

This paper introduces a cable-driven parallel robot equipped with tension sensors at its distal end. The robot demonstrated good performance in a series of palpation tests, achieving a 19% average error rate in force sensing and a 0.231 N root-mean-square error. The sensor presented here is integrated into a CDPR but the concept is generalizable to other types of cable-driven robots. The proposed design offers several key advantages:

1. Over-the-Scope Configuration: The presence of a 9 mm diameter channel at its center enables the sensor array to be used in an over-the-scope configuration. The design is also scalable for different requirements.
2. Compact Sensor Array: The sensor array is compact, with an outer diameter of 16 mm and a length of 20 mm, which can be further miniaturized. This design allows for the sensing of tension in six cables and allows delivery in the human colon, which has an average diameter range between 30 and 80 mm. Existing commercial over-the-scope devices used in the gastrointestinal tract have a larger outer diameter and length (e.g., Ovesco Colonic FTRD^®^ at 21 mm OD, 37 mm length).
3. Placement at the Distal End: By allowing placement of the sensor array at the distal end of the robot, the readings are less affected by tendon–sheath friction. This placement ensures more accurate and reliable data collection.
4. Friction Compensation: We have demonstrated that after compensating for friction, the sensor can provide readings with even higher precision, which can significantly enhance the performance and accuracy of the robot for sensing tissue stiffness and performing palpation tasks.

However, there are also some limitations: The external wrench sensing performance of the x and y axes is not as good as the z-axis, which may be due to differences in the symmetry of the cable arrangement. This force compensation strategy is currently more suitable for uniform motion. When the speed changes significantly, especially during direction changes, it can cause a large sudden shift in the external force. This could be witnessed in the blind tests, where the force feedback obtained through the haptic device was more reliable when the robot was operated at uniform speed. Potential solutions to this limitation include using a mathematical model to consider the velocity of the robot and handle rapid changes.

Ongoing work focuses on integrating the sensors and endoscope with a softer version of the robot [23] for enhancing its adaptability and safety in clinical applications. Reliable force sensing and haptic feedback could be particularly beneficial for automated surgical maneuvers that require closed-loop control, delicate tissue manipulation, and smart tissue retraction, among other applications. This could lead to more precise and safer endoscopic procedures.

## Figures and Tables

**Table 1:** Results of palpating three regions of different stiffnesses (unit: N).

Region	Black		Red		Yellow	
	1	2	3	4	5	6
Non-compensated	4.72	4.92	4.61	5.05	4.40	4.48
Double Compensated	1.18	1.21	2.79	2.74	1.59	1.50
Ground truth (F/T sensor)	1.02	1.00	2.32	2.21	1.44	1.41

**Table 2:** Force measurement performance of cable-driven surgical robots.

Robot Setup	Error Description	Measured Force Range
Tension sensor array with TSM (this work)	Average error: z-axis: 0.173 N, 0.213 N RMSE. x, y-axes: 0.268 N, 0.321 N RMSE.	0–4 N
Manipulator equipped on the DaVinci instrument base [16]	The force sensitivities are 0.2 and 0.6 N for using 1 and 2 DoF image acquisition methods, respectively.	0–3 N
Multiple-DOF cable-driven instruments [17]	0.4 N maximum error, 0.03 N signal noise, 0.05 N drift.	0–5 N (max 5 N in testing)
Flexible endoscopic robotic platform with TSM [28].	Mean RMSE 0.1711 N. Maximum error range 0.3 N to 0.5 N.	0–12 N
Experimental setup with TSM [29]	RMSE 0.0759 N, maximum error 0.1765 N.	0–2 N
Encapsulated force-sensing device in flexible robotic endoscopes [30].	Average error ~1.5 N. (Not provided in the paper, estimated based on figure results).	0–6 N

**Table 3:** Results of blind test.

Participant	1	2	3	4	5	6	7	8	9	10	Ave	STD
Black	10	9	9	10	10	8	10	9	9	10	9.4	0.70
Red	6	7	7	7	6	5	7	7	7	8	6.7	0.82
Yellow	4	6	6	4	4	3	4	5	4	5	4.5	0.97
White	1	3	3	2	1	1	1	2	2	3	1.9	0.80

## Data Availability

The data presented in this study is available on request from the corresponding author.
